# The tumour–stromal interaction between intratumoral c-Met and stromal hepatocyte growth factor associated with tumour growth and prognosis in non-small-cell lung cancer patients

**DOI:** 10.1038/sj.bjc.6601718

**Published:** 2004-03-23

**Authors:** D Masuya, C Huang, D Liu, T Nakashima, K Kameyama, R Haba, M Ueno, H Yokomise

**Affiliations:** 1 Second Department of Surgery, Kagawa Medical University, 1750-1 Miki-cho, Kita-gun, Kagawa 761-0793, Japan; 2 Department of Pathology, Kagawa Medical University, Kagawa, Japan; 3 Department of Pathology and Host Defense, Kagawa Medical University, Kagawa, Japan

**Keywords:** HGF, c-Met, Ki-67, immunohistochemistry, lung cancer

## Abstract

Immunohistochemical analyses of the effects of hepatocyte growth factor (HGF) and c-Met expression on tumour growth and angiogenesis were performed on 88 patients with non-small-cell lung cancers (NSCLCs). In all, 22 carcinomas (25.0%) were intratumoral HGF-positive, 14 carcinomas (15.9%) were stromal HGF-positive, and 36 carcinomas (40.9%) were intratumoral c-Met-positive. None of the carcinomas were stromal c-Met-positive. Examination of tumour growth revealed that the frequency of tumours with a high Ki-67 index was significantly greater for stromal HGF-positive tumours than for stromal HGF-negative tumours (*P*=0.0197). The frequency of tumours with a high Ki-67 index was also significantly greater for intratumoral c-Met-positive tumours than for intratumoral c-Met-negative tumours (*P*=0.0301). However, there was no significant difference in tumour vascularity with relation to intratumoral HGF status, stromal HGF status, and intratumoral c-Met status. The survival rate of patients with intratumoral c-Met-positive tumours was significantly lower than for patients with c-Met-negative tumours (*P*=0.0095). Furthermore, the survival rate of patients with both intratumoral c-Met-positive and stromal HGF-positive tumours was significantly lower than for patients with either positive tumours, and that of patients with both negative tumours (*P*=0.0183 and *P*=0.0011, respectively). A univariate analysis revealed that intratumoral c-Met expression was a significant prognostic factor of NSCLC patients (relative risk=2.642, *P*=0.0029). The present study demonstrates that tumour–stromal interaction between tumour cell-derived c-Met and stromal cell-derived HGF affects tumour growth and the prognosis of NSCLC patients.

Non-small-cell lung cancer (NSCLC) is one of the most common human malignancies with a poor prognosis. It is widely accepted that malignant tumours are caused by the accumulation of genetic alterations, which reflect the biological behaviour of tumours, such as aggressive cell proliferation as well as invasive and metastatic potential (Cordon-Cardo, 1995). Therefore, it is considered important to understand the biological behaviour of NSCLCs, to improve the clinical outcome of NSCLC patients.

Tumour–stromal interaction is an essential part of malignant progression *in vivo* (Chung, 1995). During tumour development, stromal fibroblasts produce an extracellular matrix that is used as an anchorage by tumour cells. In addition, the extracellular matrix also functions as a reservoir of growth factors derived from tumour or stromal cells. Various growth factors and their receptors, including hepatocyte growth factor (HGF)/c-Met, epithelial growth factor (EGF)/EGF-R, and the vascular endothelial growth factor (VEGF) family/VEGF-Rs, are reported to be involved in tumour–stromal interactions (Nakamura *et al*, 1997; Turkeri *et al*, 1998; Kajita *et al*, 2001).

Among these growth factors and their receptors, the HGF/c-Met pathway has multiple biological functions, such as cell proliferation (Montesano *et al*, 1991), motility (Weidner *et al*, 1990), angiogenesis (Bussolino *et al*, 1992), and morphogenesis (Brinkmann *et al*, 1995). Many human cancers exhibit overexpression of HGF and/or c-Met (Olivero *et al*, 1996; Kurimoto *et al*, 1998; Edakuni *et al*, 2001), and several clinical studies revealed that overexpression of HGF and/or c-Met is associated with the prognosis of NSCLC patients (Ichimura *et al*, 1996; Takanami *et al*, 1996; Siegfried *et al*, 1997). However, the mechanisms of their biological behavior in NSCLCs are not fully understood in part because they have multiple functions.

To clarify the role of HGF/c-Met in NSCLCs, we undertook a clinical study of HGF and c-Met expression in relation to tumour growth and vascularity. We evaluated their expression using immunohistochemistry to differentiate tumour cell-derived expression from stromal cell-derived expression. In addition, we studied their effects on cell proliferation rate using the Ki-67 labeling index (Gerde *et al*, 1984; Scagliotti *et al*, 1993) and their ability to promote tumour angiogenesis was evaluated by intratumoral microvessel density (IMD) using CD34 staining (Matsuyama *et al*, 1998).

## MATERIALS AND METHODS

### Clinical characteristics of patients

NSCLC patients who underwent surgery at the Second Department of Surgery, Kagawa Medical University, from January 1993 to March 2001, were examined. Tumour-node-metastasis (TNM) staging designations were assigned according to the postsurgical pathological international staging system (Mountain, 1997). Since Stage IV-lung cancer involves several ill-defined factors and has distant metastases, patients with these signs were excluded from the study. Patients with two or more types of cancers and patients, who died of causes other than NSCLC, were also excluded. In total, 88 NSCLC patients were investigated. Among them were 46 patients with adenocarcinoma, 29 patients with squamous cell carcinoma, and 13 patients with large-cell carcinoma. Patients' clinical records and histopathological diagnoses were fully documented. This report includes follow-up data until May 27, 2003. The mean follow-up period for all patients was 49.8±36.1 months.

### Immunohistochemical staining of HGF, c-Met, Ki-67, and CD34

We used a rabbit polyclonal antibody against HGF (SC-7949, Santa Cruz Biotechnology Inc., Santa Cruz, CA, USA) at 1 : 100 dilution, a rabbit polyclonal antibody against c-Met (SC-10, Santa Cruz Biotechnology Inc., Santa Cruz, CA, USA) at 1 : 100 dilution, a mouse monoclonal antibody against Ki-67 (MIB-1, DAKO, Glostrup, Denmark) at 1 : 40 dilution, and a mouse monoclonal antibody against CD34 (NU-4A1, Nichirei Corporation, Tokyo, Japan) at 1 : 10 dilution.

Formalin-fixed paraffin-embedded tissue was cut into 4-*μ*m-thick sections and mounted on poly-L-lysine-coated slides. Sections were then deparaffinized and rehydrated, heated in a microwave for 10 min in a 10-*μ*mol l^−1^ citrate buffer solution at pH 6.0, and cooled to room temperature for 20 min. After quenching endogenous peroxidase activity with 0.3% H_2_O_2_ (in absolute methanol) for 30 min, the sections were treated for 2 h at room temperature with 5% bovine serum albumin to block nonspecific staining. The sections were subsequently incubated overnight with primary specific antibodies against HGF, c-Met, Ki-67, and CD34, respectively. The slides were then incubated for 1 h with biotinylated anti-rabbit IgG (Vector Laboratories Inc., Burlingame, CA, USA) against HGF and c-Met, and biotinylated anti-mouse IgG (Vector Laboratories Inc., Burlingame, CA, USA) against Ki-67 and CD34. The sections were incubated with the avidin–biotin–peroxidase complex (Vector Laboratories Inc.) for 1 h, and antibody binding was visualized with 3,3′-diaminobenzidine tetrahydrochloride. As a final step, the sections were counterstained with Mayer's haematoxylin. Sections of resected lung tumours known to express HGF and c-Met were used as positive controls for immunostaining, and sections incubated with normal rabbit IgG served as negative reaction controls for staining of HGF and c-Met.

All immunostained sections were reviewed by two pathologists (RH and MU) who had no knowledge of the patients' clinical status. Cases with discrepancies were jointly reevaluated and a consensus was reached. In cases with multiple areas of low intensity that occurred during evaluation of immunostaining of HGF and c-Met, five areas were selected at random and scored. Also, one random field was selected in sections where all staining appeared intense. At least 200 tumour cells were scored per × 40 field. The sample was classified as intratumoral HGF-positive when ⩾50% of the tumour cells in a given specimen were positively stained for HGF, and it was classified as intratumoral HGF-negative when <50% of the cells were stained. In addition, the sample was classified as stromal HGF-positive when ⩾50% of the stromal cells of tumours in a given specimen were positively stained for HGF, and it was classified as stromal HGF-negative when <50% of the stromal cells were stained.

Since a homogeneous cytoplasmic staining pattern appeared in c-Met-stained tumour cells, c-Met staining was scored by staining intensity as reported previously (Jin *et al*, 1997; Ramirez *et al*, 2000). Staining intensity was classified as grade 0 (no staining), grade 1 (weak staining), grade 2 (moderately strong staining), grade 3 (very strong staining), or grade 4 (extremely strong staining). The sample was classified as intratumoral c-Met-positive when the intensity of c-Met-stained tumour cells in a given specimen was greater than grade 1. All other samples of c-Met-stained tumour cells were classified as intratumoral c-Met-negative.

The rate of tumour proliferation was evaluated by the percentage of carcinoma cells that stained positive for Ki-67 in a given specimen scored using the Ki-67 proliferation index. Tumours with a Ki-67 proliferation index ⩾25% were classified as high Ki-67, while tumours with <25% were classified as low Ki-67. For microvessel quantification, the three most vascularised areas detected by CD34 immunostaining were initially selected under × 40 field, and × 200 field (0.785 mm^2^ per field), and microvessels were counted in each of these areas. The average count for three × 200 fields was recorded as the IMD. Tumours with IMD ⩾90 were classified as hypervascular, while tumours with IMD <90 were classified as hypovascular (Masuya *et al*, 2001).

### Statistical analysis

The overall cancer-specific survival was defined from the date of operation to the date of cancer-related death. Statistical significances in the expression of HGF, c-Met, Ki-67, and IMD in relation to several clinical and pathologic parameters were assessed using a *t-*test and *χ*^2^ test. The Kaplan–Meier method was used to estimate the probability of overall survival as a function of time, and survival periods were compared using a log-rank test. Analysis using the Cox regression model was performed to study the effects of different variables on survival rate. All *P*-values were based on two-tailed statistical analysis, and *P*-values <0.05 were taken to indicate the statistical significance.

## RESULTS

### Hepatocyte growth factor expression in NSCLCs

Hepatocyte growth factor staining of tumour or stromal cells appeared in the form of a heterogeneous cytoplasmic staining pattern. Among the 88 carcinomas examined for HGF expression in tumour cells, 22 carcinomas (25.0%) were intratumoral HGF-positive, and 66 carcinomas (75.0%) were intratumoral HGF-negative (Table 1

**Table 1:** Distribution of 88 non-small-cell lung cancer patients according to HGF and c-Met status

		**Intratumoral HGF**			**Stromal HGF**			**Intratumoral c-Met**		
**Variables**	***n***	**Positive**	**Negative**	***P*-value**	**Positive**	**Negative**	***P*-value**	**Positive**	**Negative**	***P*-value**
*Tumor status*										
T1	35	11	24	0.2577	7	28	0.3939	9	26	0.0185
T2, T3, T4	53	11	42		7	46		27	26	

*Nodal status*										
N0	56	17	39	0.1247	10	46	0.5086	21	35	0.3895
N1, N2, N3	32	5	27		4	28		15	17	

*Pathological stage*										
Stage I	46	15	31	0.0756	9	37	0.7416	14	32	0.0169
Stage II	10	1	9		1	9		2	8	
Stage IIIA	12	0	12		2	10		8	4	
Stage IIIB	20	6	14		2	18		12	8	

*Differentiation*										
Well	30	8	22	0.0717	2	28	0.1606	10	20	0.2299
Moderately	30	11	19		5	25		16	14	
Poorly	28	3	25		7	21		10	18	

*Histology*										
Adenocarcinoma	46	16	30	0.0687	5	41	0.0520	22	24	0.1099
Squamous cell carcinoma	29	5	24		4	25		12	17	
Large-cell carcinoma	13	1	12		5	8		2	11	
										
Total number of patients	88	22	66		14	74		36	52	

HGF=hepatocyte growth factor.

and Figure 1A

**Figure 1:**
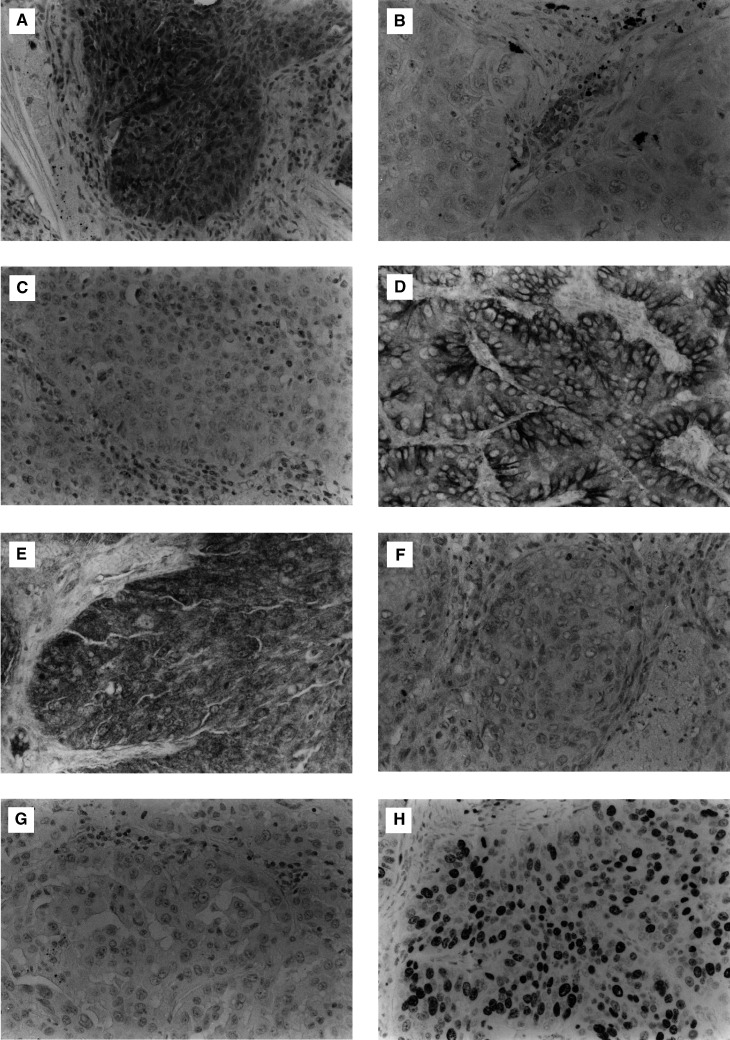
Immunohistochemical staining of human non-small-cell lung cancer tissues using the avidin–biotin–peroxidase complex procedure (original magnification, × 100). **(A)** An intratumoral HGF-positive squamous cell carcinoma. (**B**) A stromal HGF-positive squamous cell carcioma. (**C**) A stromal HGF-negative adenocarcinoma. (**D**) An intratumoral c-Met-positive adenocarcinoma. (**E**) An intratumoral c-Met-positive squamous cell carcinoma. (**F**) An intratumoral c-Met-negative squamous cell carcinoma. (**G**) An intratumoral c-Met-negative adenocarcinoma. (**H**) Ki-67 staining of an adenocarcinoma.

). There was no significant difference in intratumoral HGF expression according to tumour histology, tumour status, nodal status, and tumour differentiation. With regard to HGF expression in the stromal cells of tumours, 14 carcinomas (15.9%) were stromal HGF-positive and 74 carcinomas (84.1%) were stromal HGF-negative (Table 1 and Figure 1B, C). There was also no significant difference in stromal HGF expression according to tumour histology, tumour status, nodal status, and tumour differentiation. In addition, there was no correlation between the percentage of HGF-positive tumour cells and HGF-positive stromal cells in each NSCLC (*r*=0.066, *P*=0.5431).

### c-Met expression in NSCLCs

c-Met-stained tumour cells showed a homogeneous cytoplasmic staining pattern with variable intensity. In contrast, no carcinoma exhibited positive c-Met staining in stromal cells. Of the 88 carcinomas studied, 36 carcinomas (40.9%) were intratumoral c-Met-positive, and 52 carcinomas (59.1%) were intratumoral c-Met-negative (Table 1 and Figure 1D–G). There was no significant difference in intratumoral c-Met expression according to tumour histology, tumour differentiation, and nodal status. However, the frequency of intratumoral c-Met-positive tumours was significantly higher for T2–4 tumours than for T1 tumours (50.9 *vs* 25.7%, *P*=0.0185).

### Ki-67 proliferation index in NSCLCs

The mean value of the Ki-67 proliferation index among the 88 NSCLCs studied was 44.2±31.0. In all, 44 carcinomas (50.0%) had a high Ki-67 index and 44 carcinomas (50.0%) had a low Ki-67 index (Figure 1H). Of the 46 adenocarcinomas, 16 tumours (34.8%) had a high Ki-67 index and 22 tumours (75.9%) of the 29 squamous cell carcinomas had a high Ki-67 index. Among the 13 large cell carcinomas, six tumours (46.2%) had a high Ki-67 index. The frequency of tumours with a high Ki-67 index was significantly greater for squamous cell carcinomas than for adenocarcinomas (*P*<0.001).

### Tumour vascularity in NSCLCs

The mean IMD value in the 88 NSCLCs was 97.7±52.8. In total, 45 carcinomas (51.1%) were hypervascular and 43 carcinomas (48.9%) were hypovascular. Of the 46 adenocarcinomas 31 tumours (67.4%) were hypervascular, and eight tumours (27.6%) among the 29 squamous cell carcinomas were hypervascular. Also, six tumours (46.2%) of the 13 large-cell carcinomas were hypervascular. The frequency of hypervascular tumours was significantly higher for adenocarcinomas than for squamous cell carcinomas (*P*<0.001).

### Ki-67 proliferation index in relation to HGF and c-Met status

**Figure 2:**
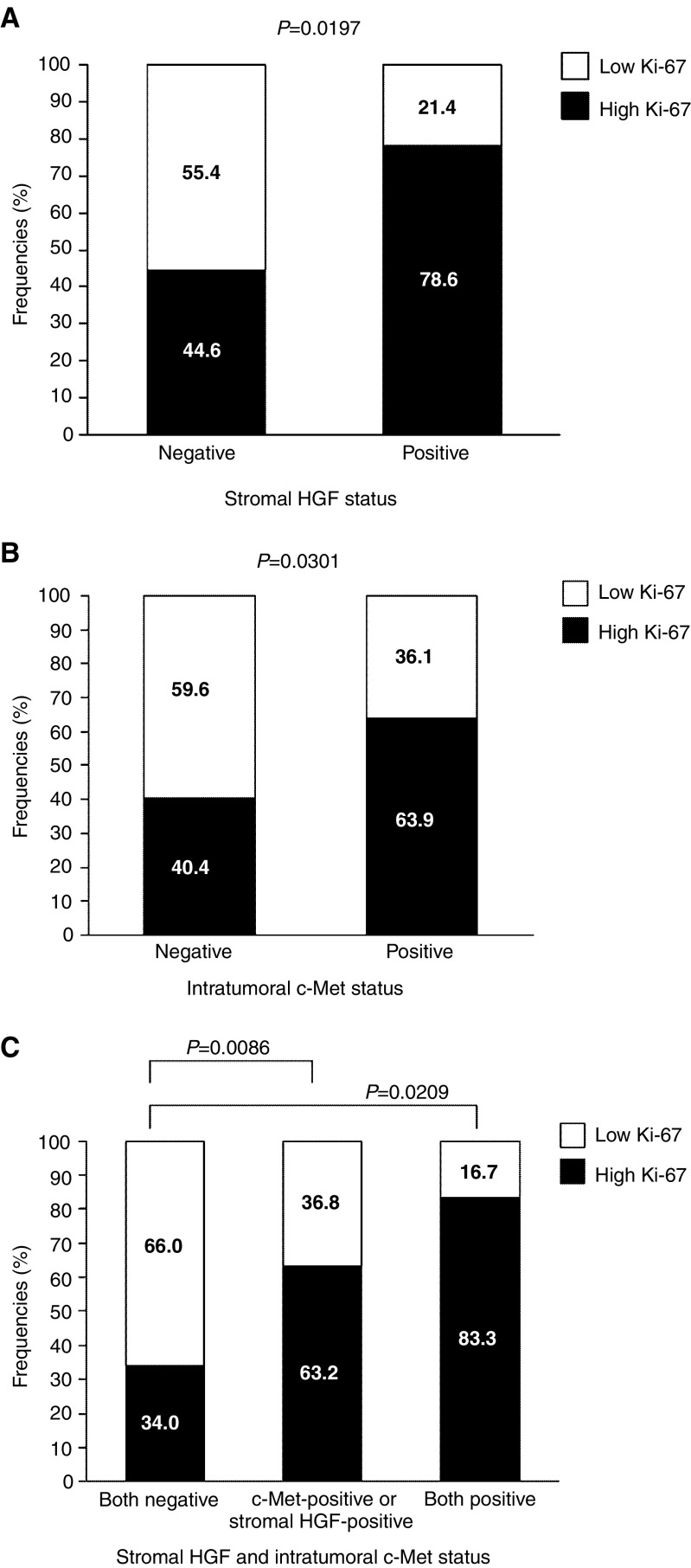
(**A**) Tumour proliferation rate in relation to stromal HGF status in NSCLCs. (**B**) Tumour proliferation rate in relation to intratumoral c-Met status in NSCLCs. (**C**) Tumour proliferation rate in relation to stromal HGF status and intratumoral c-Met status in NSCLCs.

There was no difference in Ki-67 index between intratumoral HGF-positive tumours and intratumoral HGF-negative tumours (44.7±30.8 *vs* 42.5±32.4) with regard to intratumoral HGF expression. However, the Ki-67 proliferation index was 59.9±24.5 among stromal HGF-positive tumours, and 41.2±31.4 among stromal HGF-negative tumours. The Ki-67 proliferation index was significantly greater in stromal HGF-positive tumours than in stromal HGF-negative tumours (*P*=0.0386). Of the 14 stromal HGF-positive tumours, 11 tumours (78.6%) had a high Ki-67 index, and 33 tumours (44.6%) among the 74 stromal HGF-negative tumours had a high Ki-67 index. The frequency of tumours with a high Ki-67 index was significantly greater for stromal HGF-positive tumours than for stromal HGF-negative tumours (*P*=0.0197, Figure 2A

).

The Ki-67 proliferation index was 48.5±28.8 among intratumoral c-Met-positive tumours, and 41.3±32.4 among intratumoral c-Met-negative tumours. Of the 36 intratumoral c-Met-positive tumours, 23 tumours (63.9%) had a high Ki-67 index, while 21 tumours (40.4%) of the 52 c-Met-negative tumours had a high Ki-67 index. The frequency of tumours with a high Ki-67 index was significantly greater for intratumoral c-Met-positive tumours than for intratumoral c-Met-negative tumours (*P*=0.0301, Figure 2B).

There was no significant correlation between the percentage of HGF-positive stromal cells and the percentage of c-Met-positive tumour cells in each NSCLC (*r*=0.030, *P*=0.7802). Therefore, the 88 NSCLCs examined were classified into three groups according to stromal HGF status and intratumoral c-Met status; one group in which six patients had tumours that exhibited both positive stromal HGF and intratumoral c-Met expression; a second group in which 38 patients had tumours which demonstrated either positive stromal HGF or intratumoral c-Met expression; and a third group where 44 patients had tumours that showed negative expression for both HGF and c-Met. The frequency of tumours with a high Ki-67 index was 83.3% in the first group, 63.2% in the second group, and 34.0% in the third group. The frequency of high Ki-67 tumours in the third group was significantly lower than that for the other two groups (*P*=0.0086 and 0.0209, respectively, Figure 2C).

### Tumour vascularity in relation to HGF and c-Met

No significant difference was found in IMD between intratumoral HGF-positive and intratumoral HGF-negative tumours (103.2±41.4 *vs* 96.0±55.9). In addition, there was no significant difference in IMD between stromal HGF-positive and stromal HGF-negative tumours (106.6±60.2 *vs* 95.9±51.4). Also, no significant difference was evident in IMD between intratumoral c-Met-positive and intratumoral c-Met-negative tumours (103.4±58.9 *vs* 89.3±41.4).

### Overall survival of NSCLC patients in relation to HGF and c-Met status

The 5-year survival rates of the 88 NSCLC patients according to intratumoral HGF status, stromal HGF status, and c-Met status are shown in Table 2

**Table 2:** Five-year survival rate of 88 non-small-cell lung cancer patients according to HGF and c-Met status

	**Intratumoral HGF**			**Stromal HGF**			**c-Met**		
**Variables**	**Positive**	**Negative**	**P-value**	**Positive**	**Negative**	**P-value**	**Positive**	**Negative**	**P-value**
*Tumor status*									
T1	80.8	69.1	0.2889	83.3	71.3	0.5920	72.9	72.5	0.7980
T2, T3, T4	40.4	35.8	0.6386	17.1	39.4	0.3220	21.0	53.2	0.0481

*Nodal status*									
N0	68.3	66.8	0.5148	66.7	67.9	0.8604	52.0	76.2	0.1146
N1, N2, N3	26.7	21.6	0.7577	0.0	24.9	0.2969	7.9	34.7	0.0817

*Pathological stage*									
Stage I	78.3	75.7	0.6677	75.0	77.5	0.6903	68.9	80.0	0.4205
Stage II	100.0	37.5	0.4440	0.0	50.0	0.5072	0.0	50.0	0.0941
Stage IIIA	0.0	9.1	>0.9999	0.0	11.1	0.1635	14.3	0.0	0.3456
Stage IIIB	0.0	27.9	0.2223	0.0	20.7	0.4443	9.3	42.9	0.3808

*Differentiation*									
Well	58.3	57.1	0.5321	50.0	59.0	0.6339	40.0	67.7	0.1269
Moderately	58.3	58.6	0.6983	33.3	61.1	0.4406	41.7	75.5	0.1184
Poorly	66.7	33.8	0.4610	57.1	31.6	0.3316	12.5	49.4	0.0599

*Histology*									
Adenocarcinoma	54.1	49.7	0.4936	75.0	49.6	0.5097	33.9	65.9	0.0399
Squamous cell carcinoma	75.0	55.8	0.3609	0.0	67.2	0.1573	41.3	71.8	0.1464
Large-cell carcinoma	100.0	33.3	0.3846	60.0	25.0	0.2568	0.0	45.5	0.0610
									
Total	60.3	48.6	0.1735	50.7	52.2	0.8409	33.8	63.2	0.0095

HGF=hepatocyte growth factor.

. There was no significant difference in survival among the patients in relation to intratumoral HGF status. In addition, there was also no significant difference with relation to stromal HGF status.

**Figure 3:**
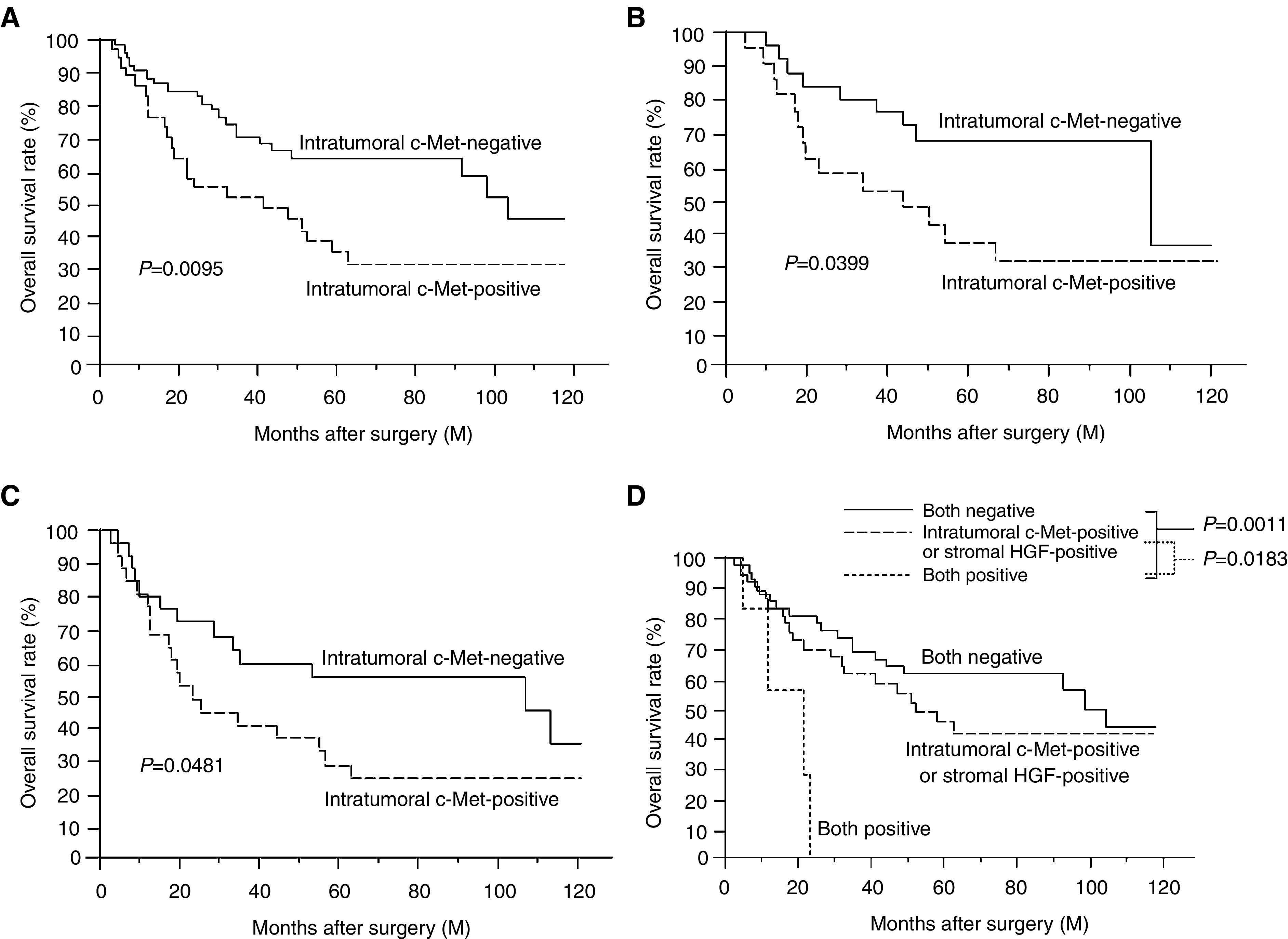
(**A**) Overall survival of 88 NSCLC patients in relation to intratumoral c-Met status. (**B**) Overall survival of 46 patients with adenocarcinomas in relation to intratumoral c-Met status. (**C**) Overall survival of 53 patients with T2–4 carcinomas in relation to intratumoral c-Met status. (**D**) Overall survival of 88 NSCLC patients in relation between stromal HGF and intratumoral c-Met status.

With respect to intratumoral c-Met status, however, the 5-year survival rate of patients with intratumoral c-Met-positive tumours was significantly lower than that for patients with intratumoral c-Met-negative tumours (33.8 *vs* 63.2%, *P*=0.0095, Figure 3A

). Also, the 5-year survival rate for patients with c-Met-positive adenocarcinomas was significantly lower than that for patients with c-Met-negative adenocarcinomas (33.9 *vs* 65.9%, *P*=0.0399, Figure 3B). Furthermore, the 5-year survival rate for patients with c-Met-positive tumours was significantly lower than that for patients with c-Met-negative tumours, especially in T2–4 tumours (21.0 *vs* 53.2%, *P*=0.0481, Figure 3C). A univariate analysis using the Cox regression model demonstrated that intratumoral c-Met status was a significant factor for predicting the prognosis of NSCLC patients (relative risk=2.642, *P*=0.0029).

Since a correlation between the Ki-67 proliferation index and both intratumoral c-Met and stromal HGF expression was demonstrated, the survival of NSCLC patients according to intratumoral c-Met and stromal HGF status was analysed (Figure 3D). The 5-year survival rate was 61.4% for patients with both negative tumours, 45.3% for patients with either type of positive tumours, and 0% for patients with both positive tumours. The 5-year survival rate for patients with both positive tumours was significantly lower than that for patients with either type of positive tumours, and that for patients with both negative tumours (*P*=0.0183 and 0.0011, respectively).

## DISCUSSION

Hepatocyte growth factor was discovered to be a mitogen for hepatocytes (Nakamura *et al*, 1989), and subsequently found to be identical to the scatter factor (SF), which destroys epithelial cell adhesion and promotes cell motility (Weidner *et al*, 1991). To date, HGF is known to be a multifunctional cytokine which induces cell proliferation (Montesano *et al*, 1991), motility (Weidner *et al*, 1990), angiogenesis (Bussolino *et al*, 1992), and morphogenesis (Brinkmann *et al*, 1995), in a wide variety of normal and neoplastic cells. In addition, its receptor is c-Met (Bottaro *et al*, 1991), a transmembrane tyrosine kinase receptor encoded by the proto-oncogene c-Met (Park *et al*, 1987).

Overexpression of HGF and/or c-Met has been reported in various human cancers, including NSCLCs (Olivero *et al*, 1996) and breast cancers (Edakuni *et al*, 2001). Some tumour cell-derived factors, such as interleukin-1, basic fibroblast growth factor, and tumour necrosis factor-*α*, are involved in the overexpression of HGF in stromal fibroblasts (Tamura *et al*, 1993; Nakamura *et al*, 1997). In addition, one study revealed that cells transformed by the ras oncogene overexpressed c-Met (Webb *et al*, 1998). Thus, such growth factors produced in stromal cells interact with the receptors expressed on tumour cells (paracrine pattern) (Chung, 1995). In addition, malignant tumour cells also often produce growth factors and their associated receptors (autocrine pattern) (Edakuni *et al*, 2001). Therefore, the HGF/c-Met pathway plays an important role during tumour progression in a paracrine pattern and/or autocrine pattern.

Several clinical studies of the HGF/c-Met pathway in NSCLCs demonstrated that its expression was associated with a poor survival rate of NSCLC patients (Ichimura *et al*, 1996; Takanami *et al*, 1996; Siegfried *et al*, 1997). However, the precise mechanisms which control their behavior in NSCLCs are still not fully understood, partly because they have a variety of function and also because they originate from tumour or stromal cells. Therefore, we undertook this study using immunohistochemistry to investigate the relationship between the HGF/c-Met pathway and both tumour growth and angiogenesis.

This study initially revealed that HGF expression appeared independently in tumour cells and/or stromal cells. In contrast, c-Met expression appeared only in tumour cells and not stromal cells, as reported by previous studies in human cancers (Ichimura *et al*, 1996; Kurimoto *et al*, 1998; Edakuni *et al*, 2001). Olivero *et al*, 1996 reported that c-Met staining was homogeneously distributed in a tumour mass, and that there was no staining of c-Met in normal lung tissue. However, HGF staining was detected in the cytoplasm of grouped cells scattered in tumour tissue, as reported previously (Olivero *et al*, 1996). These findings present here agreed well with these previous results. Therefore, we used different criteria to classify HGF and c-Met staining, respectively.

We then evaluated the rate of tumour proliferation using the Ki-67 labeling index (Gerde *et al*, 1984; Scagliotti *et al*, 1993). Ki-67 antibody recognizes the nuclear antigen expressed during G1, S, G2, and M phases of the cell cycle and not during the resting (G0) phase. The present study demonstrated significant association between the Ki-67 index and both stromal HGF and intratumoral c-Met expression. However, there was no correlation between the Ki-67 index and intratumoral HGF expression, as demonstrated by the low percentage of high Ki-67 index tumours among stromal HGF and intratumoral c-Met-negative NSCLCs. In addition, the frequency of intratumoral c-Met-positive tumours was significantly higher for T2–4 tumours than for T1 tumours. These results indicated that the interaction between stromal cell-derived HGF and tumour cell-derived c-Met promote tumour cell proliferation in a paracrine manner. To our knowledge, this study is the first clinical report on NSCLCs that demonstrates a correlation between the HGF/c-Met pathway and tumour growth through tumour–stromal interaction, as similarly reported for breast cancers (Edakuni *et al*, 2001).

The HGF/c-Met pathway is reported to be associated with angiogenesis (Bussolino *et al*, 1992), which is considered to be essential for tumour growth and metastasis (Folkman, 1990, 1995). Our previous studies revealed that tumour vascularity in NSCLCs is associated with intratumoral expression of VEGF-A (Masuya *et al*, 2001), interleukin-8 (Masuya *et al*, 2001), neural-cadherin (Nakashima *et al*, 2003), and that tumour vascularity is correlated with the survival rate of NSCLC patients (Nakashima *et al*, 2003). However, the present study did not show a correlation between the HGF/c-Met pathway and tumour vascularity in NSCLCs.

Previous clinical studies have reported that overexpression of HGF and/or c-Met is associated with the survival rate of patients with malignant tumours, including NSCLCs (Ichimura *et al*, 1996; Takanami *et al*, 1996; Siegfried *et al*, 1997), breast cancers (Edakuni *et al*, 2001), and thyroid cancers (Ramirez *et al*, 2000). However, few studies on NSCLCs have evaluated both HGF and c-Met expression, and distinguished tumour cell derived-expression from stromal cell-derived expression. The present study demonstrates that the survival rate for patients with intratumoral c-Met-positive tumours is significantly lower than that of patients with intratumoral c-Met-negative tumours, and that the survival rate for patients with tumours with both positive expression of intratumoral c-Met and stromal HGF is significantly lower than that for patients with tumours with either positive expression, or with tumours with both negative expression. Although a multivariate analysis using intratumoral c-Met status and tumour status was not proper because of tumour status depending on intratumoral c-Met expression, a univariate analysis using the Cox regression model demonstrated that intratumoral c-Met status had a significant effect on the prognosis of NSCLC patients. These results agreed with a previous clinical study on breast cancers (Edakuni *et al*, 2001).

In conclusion, the present study on NSCLCs has demonstrated that intratumoral c-Met and stromal HGF expression promote tumour growth. Furthermore, intratumoral c-Met expression is a potent prognostic factor of NSCLC patients. A recent study reported that c-Met can also be activated by semaphoring 4D to trigger invasive cell growth (Giordano *et al*, 2002). Although further studies are necessary to clarify these mechanisms (Trusolino *et al*, 2001), these studies on the HGF/c-Met pathway will aid the development of new therapeutic strategies for the treatment of NSCLC cancer patients. For example, the HGF antagonist NK4 suppresses tumour growth and could improve the clinical outcome of patients with carcinomas that exhibit overexpression of HGF and/or c-Met (Date *et al*, 1998; Kuba *et al*, 2000).

## References

[bib1] Bottaro DP, Rubin JS, Faletto DL, Chan AM-L, Kmiecik TE, Vande Wounde GF, Aaronson SA (1991) Identification of the hepatocyte growth factor receptor as the c-met prot-oncogene product. Science 251: 802–804 1846706 10.1126/science.1846706

[bib2] Brinkmann V, Foroutan H, Sachs M, Weidner KM, Birchmeier W (1995) Hepatocyte growth factor/scatter factor induces a variety of tissue-specific morphogenic programs in epithelial cells. J Cell Biol 131: 1573–1586 8522613 10.1083/jcb.131.6.1573 PMC2120656

[bib3] Bussolino F, Di Renzo MF, Ziche M, Bocchietto E, Olivero M, Naldini L, Gaudino G, Tamagnone L, Coffer A, Comoglio PM (1992) Hepatocyte growth factor is a potent angiogenic factor which stimulates endothelial cell motility and growth. J Cell Biol 119: 629–641 1383237 10.1083/jcb.119.3.629 PMC2289675

[bib4] Chung LW (1995) The role of stromal–epithelial interaction in normal and malignant growth. Cancer Surv 23: 33–42 7621472

[bib5] Cordon-Cardo C (1995) Mutation of cell cycle regulators: biological and clinical implications for human neoplasia. Am J Pathol 147: 545–560 7677168 PMC1870966

[bib6] Date K, Matsumoto K, Kuba K, Shimura H, Tanaka M, Nakamura T (1998) Inhibition of tumor growth and invasion by a four-kringle antagonist (HGF/NK4) for hepatocyte growth factor. Oncogene 17: 3045–3054 9881707 10.1038/sj.onc.1202231

[bib7] Edakuni G, Sasatomi E, Satoh T, Tokunaga O, Miyazaki K (2001) Expression of the hepatocyte growth factor/c-Met pathway is increased at the cancer front in breast carcinoma. Pathol Int 51: 172–178 11328532 10.1046/j.1440-1827.2001.01182.x

[bib8] Folkman J (1990) What is the evidence that tumors are angiogenesis dependent? J Natl Cancer Inst 82: 4–6 1688381 10.1093/jnci/82.1.4

[bib9] Folkman J (1995) Angiogenesis in cancer, vascular, rheumatoid and other disease. Nat Med 1: 27–31 7584949 10.1038/nm0195-27

[bib10] Gerde J, Lemke H, Baisch H, Wacker H, Schwab M, Stein H (1984) Cell cycle analysis of a cell-proliferation associated nuclear antigen defined by the monoclonal antibody Ki-67. J Immunol 133: 1710–1715 6206131

[bib11] Giordano S, Corso S, Conrotto P, Artigianni S, Gilestro G, Barberis D, Tamagnone L, Comoglio PM (2002) The semaphorin 4D receptor controls invasive growth by coupling with Met. Nat Cell Biol 4: 720–724 12198496 10.1038/ncb843

[bib12] Ichimura E, Maeshima A, Nakajima T, Nakamura T (1996) Expression of c-met/HGF receptor in human non-small cell lung carcinomas *in vitro* and its prognostic significance. Jpn J Cancer Res 87: 1063–1069 8957065 10.1111/j.1349-7006.1996.tb03111.x PMC5920996

[bib13] Jin L, Fuchs A, Schnitt SJ, Yao Y, Joseph A, Lamszus K, Park M, Goldberg ID, Rosen EM (1997) Expression of scatter factor and c-met receptor in benign and malignant breast tissue. Cancer 79: 749–760 9024713 10.1002/(sici)1097-0142(19970215)79:4<749::aid-cncr12>3.0.co;2-#

[bib14] Kajita T, Ohta Y, Kimura K, Tamura M, Tanaka Y, Tsunezuka Y, Oda M, Sasaki T, Watanabe G (2001) The expression of vascular endothelial growth factor C and its receptors in non-small cell lung cancer. Br J Cancer 85: 255–260 11461086 10.1054/bjoc.2001.1882 PMC2364042

[bib15] Kuba K, Matsumoto K, Date K, Shimura H, Tanaka M, Nakamura T (2000) HGF/NK4, a four-kringle antagonist of hepatocyte growth factor, is an angiogenesis inhibitor that suppresses tumor growth and metastasis in mice. Cancer Res 60: 6737–6743 11118060

[bib16] Kurimoto S, Moriyama N, Horie S, Sakai M, Kameyama S, Akimoto Y, Hirano H, Kawabe K (1998) Co-expression of hepatocyte growth factor and its receptor in human prostate cancer. Histochem J 30: 27–32 9539204 10.1023/a:1003262412346

[bib17] Masuya D, Huang C, Liu D, Kameyama K, Hayashi E, Yamauchi A, Kobayashi S, Haba R, Yokomise H (2001) The intratumoral expression of vascular endothelial growth factor and interleukin-8 associated with angiogenesis in nonsmall cell lung carcinoma patients. Cancer 92: 2628–2638 11745198 10.1002/1097-0142(20011115)92:10<2628::aid-cncr1616>3.0.co;2-f

[bib18] Matsuyama K, Chiba Y, Sasaki M, Tanaka H, Muraoka R, Tanigawa N (1998) Tumor angiogenesis as a prognostic marker in operable non-small cell lung cancer. Ann Thorac Surg 65: 1405–1409 9594875 10.1016/s0003-4975(97)01416-1

[bib19] Montesano R, Matsumoto K, Nakamura T, Orci L (1991) Identification of fibroblast-derived epithelial morphogen as hepatocyte growth factor. Cell 61: 901–908 10.1016/0092-8674(91)90363-4 1835669

[bib20] Mountain CF (1997) Revisions in the international system for staging lung cancer. Chest 111: 1710–1717 9187198 10.1378/chest.111.6.1710

[bib21] Nakamura T, Matsumoto K, Kiritoshi A, Tano Y, Nakamura T (1997) Induction of hepatocyte growth factor in fibroblasts by tumor-derived factors affects invasive growth of tumor cells: *in vitro* analysis of tumor–stromal interactions. Cancer Res 57: 3305–3313 9242465

[bib22] Nakamura T, Nishizawa T, Hagiya M, Seki T, Shimonishi M, Sugimura A, Tashiro K, Shimizu S (1989) Molecular cloning and expression of human hepatocyte growth factor. Nature 342: 440–443 2531289 10.1038/342440a0

[bib23] Nakashima T, Huang C, Liu D, Kameyama K, Masuya D, Kobayashi S, Kinoshita M, Yokomise H (2003) Neural-cadherin expression associated with angiogenesis in non-small-cell lung cancer patients. Br J Cancer 88: 1727–1733 12771988 10.1038/sj.bjc.6600955 PMC2377142

[bib24] Olivero M, Rizzo M, Madeddu R, Casadio C, Pennacchietti S, Nicotra MR, Prat M, Maggi G, Arena N, Natali PG, Comoglio PM, Di Renzo MF (1996) Overexpression and activation of hepatocyte growth factor/scatter factor in human non-small-cell lung carcinomas. Br J Cancer 74: 1862–1868 8980383 10.1038/bjc.1996.646 PMC2074802

[bib25] Park M, Dean M, Kaul K, raun MJ, onda MA, Vande Wounde G (1987) Sequence of MET proto-oncogene cDNA has features characteristic of the tyrosine kinase family of growth-factor receptors. Proc Natl Acad Sci USA 84: 6379–6383 2819873 10.1073/pnas.84.18.6379 PMC299079

[bib26] Ramirez R, Hsu D, Patel A, Fenton C, Dinauer C, Tuttle RM, Francis GL (2000) Over-expression of hepatocyte growth factor/scatter factor (HGF/SF) and the HGF/SF receptor (cMET) are associated with a high risk of metastasis and recurrence for children and young adults with papillary thyroid carcinoma. Clin Endocrinol 53: 635–644 10.1046/j.1365-2265.2000.01124.x 11106926

[bib27] Scagliotti GV, Micela M, Gubetta L, Leonardo E, Cappia S, Borasio P, Pozz E (1993) Prognostic significance of Ki67 labelling in resected non small cell lung cancer. Eur J Cancer 29A: 363–365 8398336 10.1016/0959-8049(93)90387-u

[bib28] Siegfried JM, Weissfeld LA, Singh-Kaw P, Weyant RJ, Testa JR, Landreneau RJ (1997) Association of immunoreactive hepatocyte growth factor with poor survival in resectable non-small cell cancer. Cancer Res 57: 433–439 9012470

[bib29] Takanami I, Tanana F, Hashizume T, Kikuchi K, Yamamoto Y, Yamamoto T, Kodaira S (1996) Hepatocyte growth factor and c-Met/hepatocyte growth factor receptor in pulmonary adenocarcinomas: an evaluation of their expression as prognostic markers. Oncology 53: 392–397 8784474 10.1159/000227594

[bib30] Tamura M, Arakaki N, Tsubouchi H, Takeda H, Daikuhara Y (1993) Enhancement of human hepatocyte growth factor production by interleukin-1*α* and -*β* and tumor necrosis factor-*α* by fibroblast in culture. J Biol Chem 268: 8140–8145 7681834

[bib31] Trusolino L, Bertotti A, Comoglio PM (2001) A signaling adapter function for *α*6*β*4 integrin in the control of HGF-dependent invasive growth. Cell 107: 643–654 11733063 10.1016/s0092-8674(01)00567-0

[bib32] Turkeri LN, Erton ML, Cevik I, Akdas A (1998) Impact of the expression of epidermal growth factor, transforming growth factor alpha, and epidermal growth factor receptor on the prognosis of superficial bladder cancer. Urology 51: 645–649 9586623 10.1016/s0090-4295(97)00648-1

[bib33] Webb CP, Taylor GA, Jeffers M, Fiscella M, Oskarsson M, Resau JH, Vande Wounde GF (1998) Evidence for a role of Met-HGF/SF during Ras-mediated tumorigenesis/metastasis. Oncogene 17: 2019–2025 9798673 10.1038/sj.onc.1202135

[bib34] Weidner KM, Arakaki N, Hartmann G, Vandekerckhove J, Weingart S, Rieder H, Fonatsch C, Tsubouchi H, Hishida T, Daikuhara Y, Birchmeier W (1991) Evidence for the identity of human scatter factor and human hepatocyte growth factor. Proc Natl Acad Sci USA 88: 7001–7005 1831266 10.1073/pnas.88.16.7001 PMC52221

[bib35] Weidner KM, Behrens J, Vandekerckhove J, Birchmeier W (1990) Scatter factor: molecular characteristics and effect of the invasiveness of epithelial cells. J Cell Biol 111: 2097–2108 2146276 10.1083/jcb.111.5.2097 PMC2116316

